# Fifth annual workshop of cytoreductive surgery for advanced ovarian cancer and peritoneal surface malignancies

**DOI:** 10.1186/s40661-016-0031-8

**Published:** 2016-10-24

**Authors:** Krishnansu S. Tewari

**Affiliations:** The Division of Gynecologic Oncology, University of California, Irvine Medical Center, The City Tower, 333 City Blvd W., Orange, CA 92868 USA

**Keywords:** Ovarian cancer, Cytoreduction, Workshop

## Abstract

The Fifth Annual Advanced Course in Cytoreductive Surgery for Ovarian Cancer and Peritoneal Surface Malignancies was held at and sponsored by the Division of Gynecologic Oncology at the the University of California, Irvine on Friday and Saturday, October 9-10, 2015. The workshop was comprised of didactic modules, historical treatise, an impassioned tribute, a cadaver laboratory, and heated intraperitoneal chemotherapy demonstration. This was a not-for-profit workshop, and registration fees were used to support course faculty travel to U.C. Irvine and to pay for the cadavers. The original 56 available spots were filled within three weeks of the initial announcement, prompting procurement of two additional cadavers to satisfy registration overflow and accommodate the six U.C. Irvine fellows-in-training. While international participation in the Workshops continues to rise, we have also noted more U.S.-trained Gynecologic Oncologists among the registrants.

## Introduction: ovarian cancer – the clinical problem

Cytoreductive surgery followed by adjuvant systemic platinum- and taxane-based combination chemotherapy continues to represent standard treatment of advanced ovarian cancer. Median 10-year survival rates, however, are still below 20 %, even as the optimal debulking paradigm has evolved from 1 cm^3^ residual volume of disease to that of complete resection (ie, microscopic residual or R_0_) [1]. Enthusiasm for initially promising advancements in therapeutic dosing, scheduling and route of delivery, including weekly dose-dense pacliltaxel [2, 3] and combined intraveneous/intraperitoneal chemotherapy [4, 5], has been curtailed in recent months [6–8]. Furthermore, despite eight positive, phase III, randomized trials involving five different anti-angiogenesis agents [9], vascular endothelial growth factor inhibition has only been able to significantly improve progression-free survival, not overall survival (OS). Although supporters of antivascular strategies continue to regard PFS as a valid endpoint because post-progression therapy cannot be controlled in the clinical trial setting [10], recent translational work suggests that the genomic instability which underlies ovarian carcinoma produces a phenotype comprised of different subgroups (eg., immune, *pro-angiogenic*) that limit the reach of the presumably wide net cast by anti-angiogenesis therapy [11]. The concept of oncogene addiction does not apply to this disease as prevalent driver mutations have not yet been identified [12]. Current research aims to identify and exploit somatic and germline homologous recombination deficiency mutations (eg, *BRCA1*, *BRCA2*, *RAD51*, etc.) via the synthetic lethality conferred through poly-ADP-ribose polymerase I inhibition [13–20]. In addition, strategies to break immune tolerance using programmed cell death ligand 1 and programmed death 1 inhibitors are being investigated [21–23].

Due to the absence of validated predictive biomarkers, only clinical and prognostic biomarkers are available to inform discussions with patients. Age, FIGO stage, grade, histology, performance status, volume of residual disease following cytoreductive surgery, and possibly time to initiation of adjuvant chemotherapy and rapidity of response to systemic therapy based on serial biochemical and radiographic analyses represent powerful prognostic factors for this disease. While performance status can be modified through medical and nutritional intervention, the time to initiation of chemotherapy is dependent on multiple factors including access to care, insurance authorizations, and perioperative sequelae. Thus, the only prognostic factor which is under control of the oncologist is the volume of tumor residual following cytoreductive surgery.

## Workshop planning

**Fig. 1:**
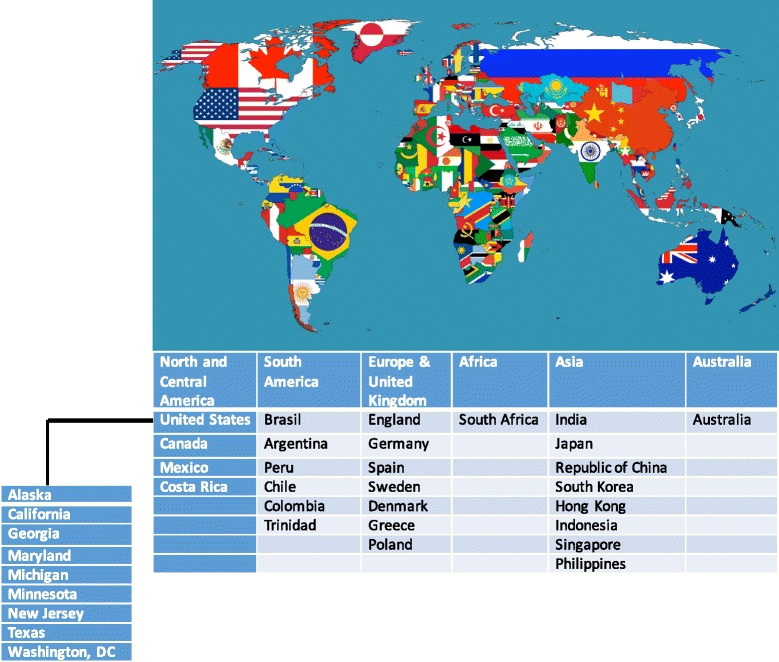
Global and U.S. Interest in surgical workshops for advanced ovarian cancer

The Fifth Annual Cytoreductive Surgery Workshop for Advanced Ovarian Carcinoma and Peritoneal Surface Malignancies was held in Orange County, California. Didactics were delivered at the Surf & Sand Hotel in Laguna Beach and cadaver dissections with demonstration of cardinal advanced cytoreductive surgical strategy were performed at the University of California, Irvine Main Campus in Irvine. The assembled faculty included course director Robert E Bristow, MD from the home institution (radical oophorectomy), Krishnansu S. Tewari, MD (also from UC Irvine; extrapelvic colon and bowel), Scott M Eisenkop from the Women’s Cancer Center in Sherman Oaks, CA (retroperitoneum), David Imagawa from UC Irvine (liver), William A. Cliby from the Mayo Clinic in Rochester, MN (spleen, diaphragm), Cyril W. Helm from St. Louis University Hospital (HIPEC), and Dennis S. Chi from Memorial Sloan-Kettering Cancer Center in New York (VATS), Similar to previous years, there was representation from the nations of six continents. Interestingly, while the international attendance which started off relatively high in 2011, continues to grow, this year there were many more attendees from the United States (Fig. 1, Table 1).

**Fig. 2:**
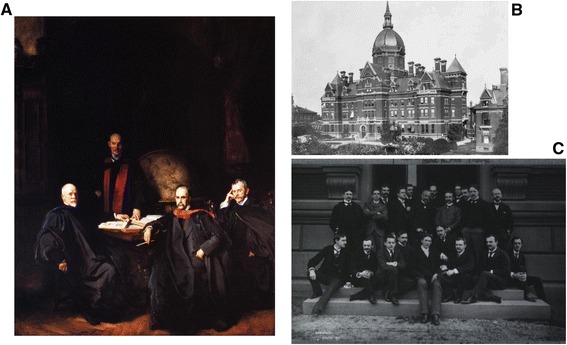
Johns Hopkins University. **a** John Singer Sargent’s The Four Doctors (1906) depicting the Founding Professors (left to right): Welch (pathologist & Dean), Osler (internist), Halstead (surgeon), and Kelly (gynecologist). **b** Early photograph of The Johns Hopkins Hospital. **c** Johns Hopkins Medical Class of 1892 with Dr(s) Kelly and Clark seated in the front row (third and second from the right, respectively). All images in the Public Domain

**Table 1:** Six years of cytoreductive surgery workshops

Year	Course site	# Attendees	# Countries represented
2011	U.C. Irvine Medical Center, Orange, CA	25	12
2012^a^	St Louis University Medical Center		
2013	U.C. Irvine Main Campus (Irvine)^b^	36	18
2014^a^	Newcastle on Tyne, United Kingdom		
2015	U.C. Irvine Main Campus (Irvine)^b^	59	26
2016^c^	Newcastle on Tyne, United Kingdom		
	Philippine Society of Gynecologic Oncology, Manila, Philippines		
	U.C. Irvine Main Campus Fellows-in-Training Course		

^a^Satellite workshops

^b^Didactics given at the Surf & Sand Hotel in Laguna Beach, CA

^c^2016 workshops in Manila and in the UK have only recently been completed and therefore final registration data concerning number of participants and demographics is not yet available; the Fellows' Workshop is not scheduled to take place until November

Didactics were subdivided into three components: 1) a history of the evolution of cytoreductive surgery including the advancement of a new thesis concerning the origins of cytoreduction for ovarian cancer (now regarded as the “2nd Law of Cytoreduction”); 2) lectures on surgical innovations and strategic planning in systematic anatomic manner based on intra-abdominal and extra-abdominal spread patterns of disease; 3) tribute to a pioneer in the field of surgery. At the 14 cadaver stations, faculty rotated among attendees to illustrate the principles of eight anatomic surgical procedures required to accomplish an R_0_ designation at completion of cytoreduction.

## Reiteration of the First Thesis

Introduced by faculty member, KS Tewari, at the 1st Workshop on November 4, 2011, the concept of cytoreduction for ovarian cancer evolved over three periods encompassing nearly 200 years [24]. Beginning with Ephraim McDowell’s (1771-1830) first oophorectomy performed on 45-year old Jane Todd Crawford (1763-1842) on his kitchen table on Christmas Day, 1809, a surgical masterpiece was created for which McDowell was named the Father of Abdominal Surgery [25]. Attempts at oophorectomy or ovarian cystectomy preceding McDowell’s time are not in short supply (including forays into antiquity and even Robert Houston’s 1701 “puncture” of an ovarian cyst [26]), but McDowell was the first to safely remove an ovarian tumor and describe his technique in detail. The second period encompassed the idea that surgery could be performed even in the setting of metastatic disease, with proponents including Joe Vincent Meigs (1892-1963) of Boston Hospital [27], Hudson’s radical oophorectomy for fixed cancers in the pelvis [28], and Hugh R. K. Barber (1918-2006) and Alexander Brunschwig (1901-1969) who used pelvic exenterative procedures (designed for women suffering from central, isolated recurrence of cervical cancer following radiotherapy) to treat 22 women with advanced and recurrent ovarian cancers [29]. The third and final period of the evolution of the concept of cytoreduction and inherent validity in advanced disease encompasses the 1973 report by McGrath on the survival advantage afforded patients with abdominal Burkitt’s lymphoma who underwent extensive disease resections [30], the landmark study by Griffiths from 1974 in which the surgeon reported that among 102 consecutive cases of advanced ovarian cancer, those patients in whom residual disease greater than 1.5 cm in maximal diameter was left in the abdominal cavity were invariably dead within 2 years as compared to the 20 % 5-year survival rate conferred by tumor residuals under 1.5 cm [31], and finally, the watershed event by Bristow et al. in which a meta-analysis of 81 ovarian cancer cohorts (nearly 7,000 patients) treated with platinum-based chemotherapy demonstrated that the most important determinant of survival was maximal cytoreduction with each 10 % increase in tumor resection associated with a 5.5 % increase in median survival time [32]. This chronology appears in greater detail in our first Workshop Report [24]. The First Law of Cytoreduction is concerned with the temporal aspects and holds that the evolution traversed three distinct periods involving oophorectomy, surgery in the setting of metastatic disease, and validation of surgical effort to bring the residual disease volume to no visible tumor (ie, R_0_).

## The Road to Baltimore (New Thesis or 2nd Law)

The rationale for cytoreduction is supported by several hypotheses concerning intrinsic tumor drug resistance (which is independently described by both the Goldie Coldman hypothesis for acquisition of somatic mutations and Bayes Theorem) by which large areas of disease harboring chemoresistant clones are resected [24]. Increased tumor growth fraction (described according to Gompertzian cell kinetics) occurring after surgery shuttles resting phase G_0_ cells into the cell cycle and thereby makes them more vulnerable to cycle-specific antineoplastic agents. Maximal cytoreduction also results in increased drug perfusion, a lower likelihood of developing acquired drug resistance, and enhanced host immunologic competence. To date the best outcomes among women with advanced ovarian cancer appear to be in the group of patients who can withstand cytoreductive surgery to optimal disease status (ie., low volume residual < 1 cm or R_0_) and six cycles of combined intraperitoneal-intravenous chemotherapy.

Optimal cytoreduction and/or complete resection in the abdomen and pelvis is accomplished through radical pelvic surgery and in many cases upper abdominal procedures with complete parietal and visceral peritonectomy [33]. Interestingly, radical pelvic surgery was initially applied to cervical cancer. Although surgeons in Germany (A. K. Mackenrodt (1859-1925)) and in Austria (eg., Frederick Schauta (1849-1919), vaginal approach), Ernst Wertheim (1864-1920, abdominal approach) were developing the technique of radical hysterectomy for cervical cancer during the latter part of the 19th Century [34], it was in the United States, specifically in Baltimore, Maryland, that a formal treatise was prepared.

The Johns Hopkins University was founded in 1876 and named after its first benefactor the American entrepreneur, abolitionist, and philanthropist, Johns Hopkins. With the completion of Johns Hopkins Hospital in 1889 and the medical school in 1893, the university’s research focus attracted faculty members with international reputations who would ultimately emerge as major figures in academic medicine. Among the “Big Four” founding professors of Johns Hopkins Hospital (Fig. 2a-b) was Sir William Osler, 1st Baronet (1849-1919), the internist who would bring medical students out of the lecture hall for bedside clinical training and become known as the “Father of Modern Medicine”. William Henry Welch (1850-1934) was the pathologist (and bacteriologist) and served as the first Dean of the Johns Hopkins School of Medicine. William Stewart Halsted (1852-1922) was the surgeon who emphasized strict aseptic technique and developed the *en bloc* radical mastectomy for breast cancer [35]. Finally, Howard Atwood Kelly (1858-1943) was the gynecologist who had been trained by James Marion Sims (1813-1883), the “Father of Modern Gynecology” who developed the Sims speculum and the surgical technique for repair of vesicovaginal fistula due to obstructed childbirth. Kelly himself is credited for having established gynecology as a distinct specialty and developing new surgical instruments and surgical approaches to gynecologic diseases.

**Fig. 3:**
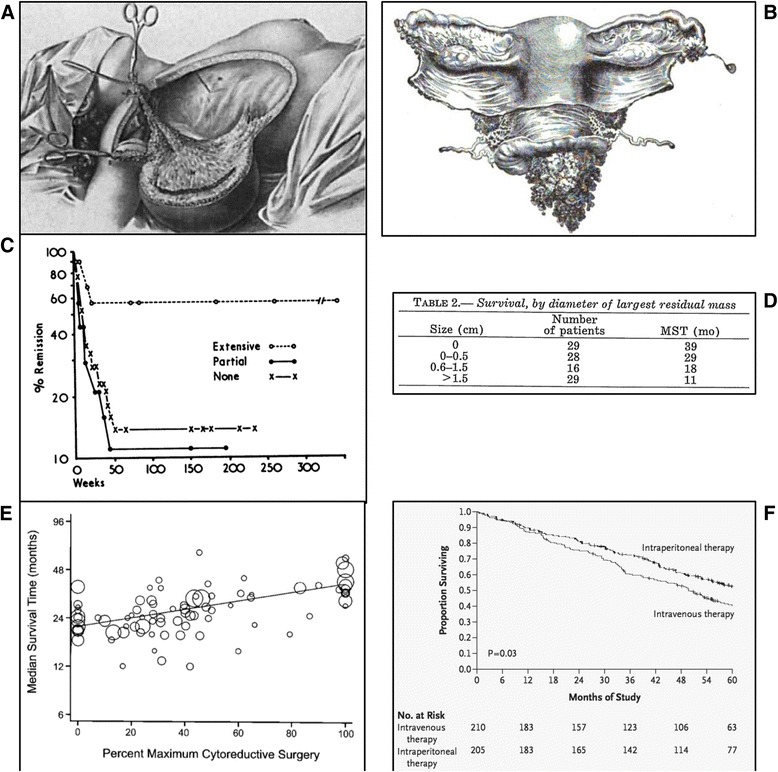
Evolution of *en bloc* and cytoreductive surgery. **a** Halsteadian principle of radical mastectomy [35]; **b** Clark’s radical hysterectomy for carcinoma of the cervix [37]. **c** Magrath’s demonstration of improved survival with surgical resection of abdominal Burkitt’s lymphoma [30]; **d** Original table from Griffith’s seminal report on the impact of residual disease following surgery and survival in ovarian cancer [31]; **e** Bristow’s meta-analysis demonstrating that each additional 10 % effort at maximal cytoreduction confers a 5.5 % survival advantage in advanced ovarian cancer treated during the platinum era [32]; **f** Overall survival curves from GOG 172 demonstrating 16 month improvement among women who had undergone optimal cytoreduction followed by intravenous-intraperitoneal chemotherapy [4]

John Goodrich Clark (1867-1927) (Fig. 2c) had trained at the University of Pennsylvania and interned at a local Philadelphia hospital before coming to Johns Hopkins. He had originally been granted a residency position with Osler, but upon arrival he was told that that position had been committed to another physician. Fortunately, Kelly had an opening, and so through a quirk of fate, Clark changed career paths abruptly from internal medicine to gynecology [34]. Kelly assigned Clark the task of developing a more radical approach to the treatment of cervical cancer. At pathologic examination, Clark noted that in 15 of 20 cases, the disease had extended beyond the margins of resection. Having become influenced by the surgical doctrines of Halstead (Fig. 3a), Clark began considering the application of Halsteadian principles to *en bloc* radical hystererctomy for cervical cancer [34] (Fig. 3b). The difference between Clark and his more senior European contemporaries (eg., Mackenrodt, Schauta, Wertheim), was in rationale, thesis, and methodology [36].

John Hopkins Hospital is where Robert E Bristow (currently Chairman of Obstetrics & Gynecology at UC Irvine Medical Center in Orange, California) began collecting data on not only the performance of radical hysterectomy with radical oophorectomy (which encompassed in most cases a low anterior resection) for advanced ovarian cancer, but the performance of numerous other upper abdominal operations required to bring the residual disease burden down to zero (eg., splenectomy, full-thickness diaphragm resection, porta hepatis surgery, supra-renal lymphadenectomy, video-assisted thorascopic surgery (VATS), etc.) [37]. As stated earlier, this work culminated in what remains a heavily cited paper in the field, specifically Bristow’s meta-analysis performed while in Baltimore and published in 2002 [32] (Fig. 3d).

Finally, John Hopkins medical oncologist, Deborah Armstrong, served as the Study Chair and Principal Investigator for GOG protocol 172, the phase III randomized trial of intravenous chemotherapy vs intravenous-intraperitoneal chemotherapy which demonstrated a 16-month OS advantage in the combined therapy arm [4] (Fig. 3f). Regional therapy exploits both the prolonged confinement of disease within the peritoneal cavity and the steep dose-response relationship observed for many cytotoxic agents, allowing for a slower rate of drug clearance from the peritoneal to systemic compartments, ultimately creating a concentration differential across the peritoneal-plasma barrier that favors the peritoneal cavity [24].

GOG 172 was actually the third phase III randomized trial by the GOG to demonstrate a survival advantage with intraperitoneal chemotherapy, but all three studies had been subject to reasonable scrutiny, with GOG 104 (published in 1996 [38], the same year that GOG 111 was published and demonstrated the superiority of combining paclitaxel with cisplatin over cyclophosphamide plus cisplatin [39]) not including taxanes, and with GOG 114 (published in 2001) [40] being criticized for intravenous platinum dose escalation in the intraperitoneal arm (ie., prior to intraperitoneal therapy patients received 2 cycles of intravenous carboplatin at AUC 9). Not only was GOG 172 marred by significant toxicity which only permitted 40 % of patients on the combined intravenous-intraperitoneal arm to receive all six cycles of adjuvant therapy, but the schedule of paclitaxel on the intravenous-intraperitoneal arm (24 h intravenous pacliltaxel (135 mg/m^2^ BSA) on day 1 followed by intraperitoneal paclitaxel (60 mg/m^2^ BSA) on day 8) suggested that weekly dose-dense taxane therapy could have accounted for the superior results. Despite these criticisms, a follow-up combined analysis of GOG 114 and GOG 172 by Tewari et al., does attest to the long-term survival (ie, 9 years) benefit of combined intravenous-intraperitoneal therapy [5]. Recent presentations at the 2016 Annual Meeting of the Society of Gynecologic Oncology (ie., GOG 252), have been disappointing with respect to intraperitoneal chemotherapy, with GOG 252 not having a proper control arm making the entire trial essentially uninterpretable [7]. Nevertheless, given the long-standing shortcomings of the three previous IV-IP vs IV phase III randomized trials described above, the negative results of GOG 252 have added to the growing disenchantment with IP therapy that has gained traction with many oncologists who treat women with advanced disease.

Criticisms aside, it is clear that John Hopkins Hospital in Baltimore has served as fertile ground for what is considered the treatment paradigm for advanced ovarian cancer. The Second Law of Cytoreduction concerns itself with geospatial constraints and holds that the development of radical pelvic surgery, together with upper abdominal procedures and adjuvant regional therapy took place at *ground zero* in Baltimore.

## Tribute to Dr. Francis D. Moore (1913-2001)

**Fig. 4:**
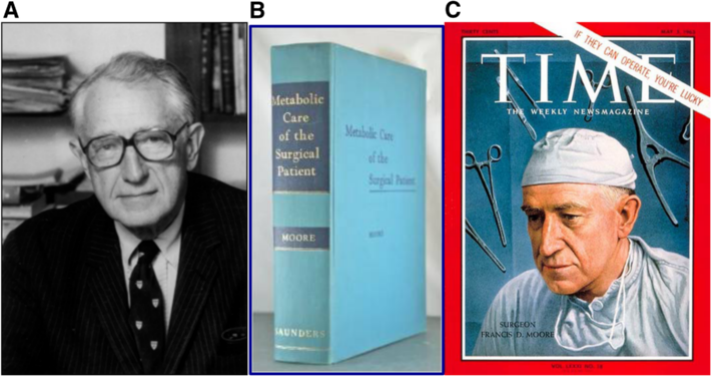
Understanding the physiologic responses to surgery has allowed surgeons to safely perform the multiple surgical procedures required at times to completely cytoreduce women with advanced ovarian cancer. **a** Dr. Francis D. Moore (1913-2001); **b**: Metabolic Care of the Surgical Patient by Moore; **b** Dr. Moore on the cover of Time Magazine, May 1963

At the 2015 Cytoreductive Surgery Workshop a tribute to Dr Francis Moore was made during the didactic component (Fig. 4a). His classic books, Metabolic Response to Surgery (1949) and Metabolic Care of the Surgical Patient (1959) (Fig. 4b), are regarded as masterpieces that according to Judah Folkman (1933-2008), the pioneer of angiogenesis and anti-angiogenesis therapy [41], changed the thinking of surgeons throughout the world and reduced suffering and mortality of their patients [42] (Fig. 4c). Before Moore, surgeons concentrated on improving their craft to effect the local anatomic changes necessary to treat disease, but they remained perplexed by the body’s physiologic response to the trauma of surgery. Surgeons during the first half of the 20th Century did not understand how to optimize the physiologic status of their patients before surgery. A perfect anatomical operation could be followed by disastrous complications or death from a low level of circulating sodium chloride or magnesium, or a high level of potassium chloride, or an undetected loss of plasma water [43]. Dr Moore was among the first translational scientists and his studies carried out between the physiology laboratory and the patient’s bedside culminated in his classic books which were regarded as the “Bible” of surgery for five generations of surgeons. The book was so well-written that in his lifetime, no updated/expanded edition needed to be written. At the 2015 Workshop, the purpose of the tribute was to recognize Moore’s trailblazing work as that fundamental body of knowledge which has provided gynecologic oncologists with the knowledge base and confidence to pursue advanced surgical resections in women with ovarian cancer (Fig. 4b).

## Surgery for Ovarian Cancer – A Surgical Atlas

The 2015 Workshop was held to coincide with publication of the 3rd edition of Surgery for Ovarian Cancer, which continues to be edited by Bristow RE, Karlan BY, and Chi DS. The 3rd edition is noteworthy for allowing access to the entire text as a VitalSource® ebook and contains instructions for creating a VitalSource Bookshelf upon redemption of the code contained on the scratch-off panel on the inside front cover. Internet links to high definition video illustrating many of the surgical procedures presented at the Workshop are also distributed throughout the text. All Workshop participants were provided a copy of this text book.

## Cadaver stations

**Fig. 5:**
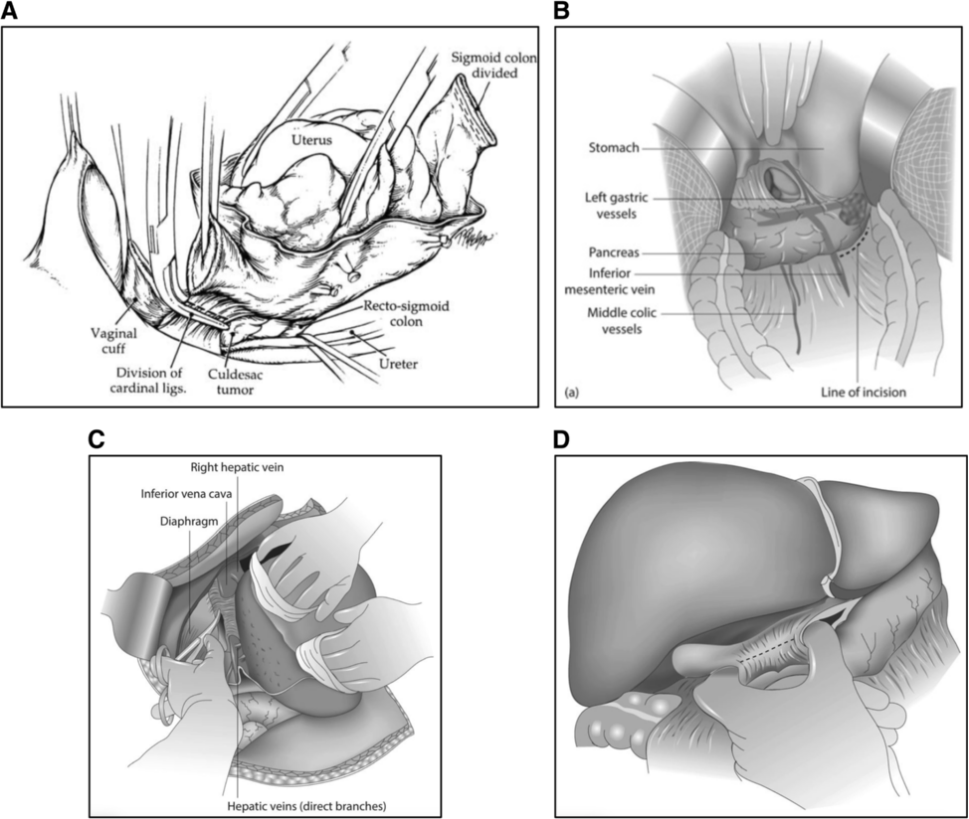
Highlights from Cadaver dissections at the 2015 Surgery Workshop: **a** radical oophorectomy; **b** splenectomy with distal pancreatectomy; **c** hepatic mobilization; **d** Pringle maneuver. Figure 4a from Bristow RE, et al. J Am Coll Surg 2003; **b**-**d** from Surgery for Ovarian Cancer, 3rd Edition, Bristow RE, Karlan BY, Chi DS (eds), Taylor & Francis Group, 2015. All images used with permission
- The integrity of the rear admiral’s circular end-to-end anastomosis is demonstrated by the production of two symmetrical, intact donuts and a negative bubble test.

Coincident with the adaptation of surgical technique within the confines of this disease along with incorporation of procedures from other disciplines, cadaver dissections continue to evolve. For example, the attendees at the 2011 workshop were notably interested in the performance of low anterior resection with end-to-end anastomosis, splenectomy, and peritoneal stripping from the diaphragm. In 2015, cadaveric stations catering to the performance of the following were most popular:

- Radical oophorectomy (key points):
- The *en bloc* specimen includes the uterus, adnexae, anterior pelvic peritoneal tumor, cul-de-sac tumor, and rectosigmoid colon, all contained within the *peritoneal bag* to leave the pelvis macroscopically tumor-free (Fig. 5a).

- Splenectomy with distal pancreatectomy (key points):
- Identification of the left gastric vessels and the inferior mesenteric vein (IMV) (Fig. 5b).
- Umbilical tape placed to the left of the IMV
- Individual ligation of the splenic artery and vein
- *En bloc* resection of distal pancreas with spleen by dividing the tail distal to ligated splenic vessels
- Distal pancreas closed with running stitch of delayed absorbable suture followed by reinforcing layer of 2-0 silk mattress stitches.

- Liver mobilization to allow full-thickness resection of the right diaphragm (key points):
- Following division of the round ligament, the falciform ligament is divided towards its apex where it bifurcates into the coronary ligament
- Incision of the coronary ligament exposes the right hepatic vein and inferior vena cava and the dissection is maintained superficial to these vessels (Fig. 5c)
- The liver is drawn inferiorly and medially by releasing the ligamentous attachments of the left lobe
- In sequence, the left triangular ligament, the anterior and posterior layers of the left coronary ligament, the hepatogastric ligament, the anterior layer of the right coronary ligament, and the right triangular ligament are divided, exposing the right paracolic gutter and Morison’s pouch.
- The incision for diaphragm resection should include a 0.5-1.0 cm margin and run parallel to the path of the branching phrenic nerve.

In addition to the stations listed above, the 2015 Workshop included cadaver dissections to demonstrate suprarenal aortic lymphadenectomy, resection of porta hepatis disease, and the Pringle maneuver in which the index finger is inserted through the foramen of Winslow and the thumb through a defect in the gastrohepatic ligament to access the porta hepatis, allowing for total inflow occlusion via an atraumatic clamp or silastic vessel loop to prevent unnecessary blood loss during wedge and/or hepatic resections (Fig. 5d). Both heated intraperitoneal chemotherapy (HIPEC) and video-assisted thorascopic surgery (VATS) were also featured in the cadaver laboratory, with the latter representing a new addition to the 2015 Workshop.

## Discussion

The discipline of surgery requires life-long learning. In the subspecialty of Gynecologic Oncology, this principle has been best exemplified by the introduction of minimally invasive surgery into the management of clinical stage I endometrial cancer, FIGO stage I cervical cancer (including lesions amenable to fertility-preserving radical trachelectomy), and select adnexal masses. Some practicing Gynecologic Oncologists had little or no laparoscopic training during fellowship, and many had no robotics training. Minimally invasive workshops held by the American Association of Gynecologic Laparoscopists and by the Society of Gynecologic Oncology in collaboration with industry partners (eg., Intuitive Surgical, Ethicon, Covidien, etc.) have helped disseminate the knowledge of minimally invasive surgery throughout the subspecialty and brought many surgeons up to speed with this essential treatment modality.

While the principles of cytoreductive surgery are taught in Gynecologic Oncology fellowship training programs throughout the world, the demonstration of technique and how to safely push the envelope to accomplish optimal debulking status or even an R_0_ resection appears to vary to some degree. In the EORTC randomized trial of primary surgery vs neoadjvuant chemotherapy, Vergote et al. reported that the hazard ratio (HR) for death in the group assigned to neoadjvuant chemotherapy followed by interval debulking surgery, (as compared with the group assigned to primary debulking surgery followed by chemotherapy), was 0.98 (90 % CI, 0.84-1.13; p-0.01 for non-inferiority) and the HR for PFS was 1.01 (90 % CI, 0.89-1.15) [44]. Although postoperative rates of adverse effects and mortality tended to be higher after primary debulking than after interval cytoreduction, this study raised a number of controversies, particularly regarding the quality of debulking surgery. While complete resection of all macroscopic disease (at primary or interval surgery) was the strongest independent variable in predicting OS, only 41.6 % of patients were rendered optimally debulked to 1 cm or less of residual tumor following primary cytoreduction, as compared to 80.6 % of patients after interval cytoreduction. Similarly, in the MRC CHORUS Trial which also demonstrated significant non-inferiority between primary and interval cytoreduction arms, only 25 % of patients in the primary surgery cohort were left with < 1 cm residual disease [45]. These results for optimal cytoreduction rates are much lower than what is reported by many U.S. centers. For these reasons it was not surprising that beginning with the First Annual Cytoreductive Surgery Workshop in 2011 that attendees were registering from around the world, with each succeeding Workshop receiving even more participation from our global partners.

**Fig. 6:**
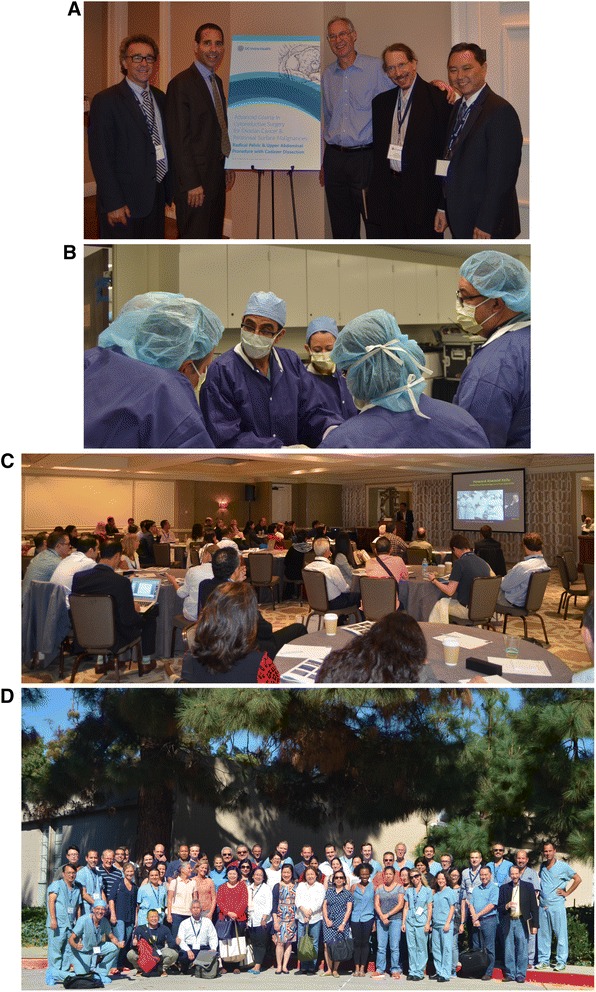
The Fifth Annual Cytoreductive Surgery Workshop (2015). **a** Workshop faculty (from left to right): Dr(s) Cliby, Bristow, Helm, Eisenkop, and Chi; **b** An astonished Ramirez Escobar attempts to dissect a cadaver in the laboratory; **c** Dr. Tewari presents his thesis, *The Road to Baltimore*, during didactics; **d** Participants (attendees, faculty, and UCI fellows) seen outside of the cadaver dissection laboratory

Interestingly, each succeeding workshop has also been met with increased registration from graduates of American Board of Obstetrics & Gynecology certified training programs. The high numbers of U.S. participants in the 2015 workshop (Fig. 6) may be the result of the aftermath following plenary presentation of GOG data at the 2015 SGO Annual Meeting in which notable discrepancies were reported between surgeon’s assessment of cytoreduction status and pre-treatment imaging on a U.S.-led phase III randomized clinical trial of anti-angiogenesis therapy for newly diagnosed advanced ovarian carcinoma [46]. Enthusiasm for these types of activities has led directly to our first Cytoreductive Surgery Workshop designed specifically for fellows-in-training. All 24 spots for this 2016 Fellows Cytoreductive Surgery Workshop were filled within two hours of its announcement.
